# Does employer involvement in primary health care enhance return to work for patients with stress-related mental disorders? a cluster randomized controlled trial

**DOI:** 10.1186/s12875-023-02151-0

**Published:** 2023-09-20

**Authors:** Anja Beno, Monica Bertilsson, Kristina Holmgren, Kristina Glise, Anders Pousette, Karin Segerfelt, Lisa Björk

**Affiliations:** 1 Institute of Stress Medicine, Region Västra Götaland, Carl Skottsbergs Gata 22B, SE-413 19 Gothenburg, Sweden; 2 School of Public Health and Community Medicine, Institute of Medicine, Sahlgrenska Academy, Gothenburg University, Gothenburg, Sweden; 3 Department of Health and Rehabilitation, Institute of Neuroscience and Physiology, The Sahlgrenska Academy at the University of Gothenburg, Gothenburg, Sweden; 4 The Department of Psychology, University of Gothenburg, Gothenburg, Sweden; 5 The Department of Work Science and Sociology, University of Gothenburg, Gothenburg, Sweden

**Keywords:** Stress-related disorders, Sick leave, Return to work, Employer

## Abstract

**Background:**

Stress-related disorders have become a major challenge for society and are associated with rising levels of sick leave. The provision of support to facilitate the return to work (RTW) for this patient group is of great importance. The aim of the present study was to evaluate whether a new systematic procedure with collaboration between general practitioners (GPs), rehabilitation coordinators (RCs) and employers could reduce sick leave days for this patient group.

**Method:**

Employed patients with stress-related diagnoses seeking care at primary health care centres (PHCCs) were included in either the intervention group (*n* = 54), following the systematic intervention procedure, or the control group (*n* = 58), receiving treatment as usual (TAU). The intervention included a) a training day for participant GPs and RCs, b) a standardised procedure for GPs and RCs to follow after training, c) the opportunity to receive clinical advice from specialist physicians in the research group. Outcome measures for RTW were sick leave days.

**Results:**

The median number of registered gross sick leave days was lower for the control group at six, 12 and 24 months after inclusion, but the difference was not statistically significant. The control group had significantly fewer net sick leave days at three months (*p* = 0.03) at six months (*p* = 0.00) and at 12-months follow-up (*p* = 0.01). At 24 months, this difference was no longer significant.

**Conclusions:**

The PRIMA intervention, which applied a standardized procedure for employer involvement in the rehabilitation process for patients with stress-related disorders, actually increased time to RTW compared to TAU. However, at 24 months, the benefit of TAU could no longer be confirmed. The study was registered on 16/01/2017 (ClinicalTrials.gov, NCT03022760).

**Supplementary Information:**

The online version contains supplementary material available at 10.1186/s12875-023-02151-0.

## Background

During the last decade, common mental disorders are the most prevalent diagnoses leading to sick leave among Swedish workers. Within this group, stress-related mental disorders were the most common diagnoses, including acute stress reaction, reaction to severe stress and stress-related exhaustion disorder (ED) [1]. In January 2022, 20.3% of all sick leave cases in Sweden had stress-related diagnoses [2]. Increased sick leave due to work-related stress is also a general trend seen across Europe [3].

The consequences of stress-related disorders can be found at all levels: for individuals, who often have long-lasting symptoms and sick leave; for employers, due to loss of productivity and high levels of absenteeism and turnover; and for society, due to increased costs for social security and health care [4–6].

Work-related conditions often play an important role, and factors such as high demands and low control, high workload, low reward, and job insecurity increase the risk of developing stress-related symptoms [7]. The consequences of stress differ and can lead to a broad spectrum of symptoms, where acute stress reactions [8] have a good prognosis and, in some cases, sick leave is necessary for a short period. Long-term stress can lead to diagnoses such as reaction to severe stress, burnout [8] or exhaustion disorder (ED) [9]. Previous studies have found that symptoms resulting from long-lasting stress exposure, such as mental exhaustion, cognitive dysfunction and reduced stress tolerance, tend to remain for a long time [10–13]. These symptoms significantly impact perceived work ability, consequently affecting work productivity [14]. Long-term sick leave is sometimes necessary, and return to work (RTW) can take several years [13]. It is of great interest to promote RTW for patients with stress-related diagnoses, however, it is challenging to draw conclusions from the available evidence since the methods used to define and measure RTW and mental disorders, including stress-related diagnoses, differ between studies. Another aspect is that health care, occupational health and safety, and social security systems differ between countries.

Several systematic reviews on the effect of RTW interventions in people with common mental disorders (CMD), including work-related stress, have been conducted and have shown ambiguous [15–17] or relatively weak effects [18, 19]. However, Mikkelsen et al. [20] found that contact with the workplace is an important factor for a successful intervention, particularly when it comes to interventions targeting stress. In a broad review of work-focused interventions in primary care, Reed and Kalaga [21] identified time constraints, poor access to rehabilitation services and poor coordination between general practitioners (GPs) and occupational physicians as important barriers for patients with mental disorders to obtain, retain or return to employment. Another obstacle relates to the difficulties GPs face when trying to determine the effects of being away from work on their patients’ symptoms, well-being and recovery. Physicians play an important role by gathering information about the person, the work and home environment and work tasks/occupations [22]. The competence of the individual GP is also important for the treatment of patients with stress-related disorders [23].

There are, however, recent examples of promising interventions in clinical settings. In a non-controlled clinical trial consisting of a 24-week multimodal intervention at two specialised rehabilitation centres in Sweden, including sessions with rehabilitation coordinators and rehabilitation meetings for patients with stress-induced exhaustion disorder, researchers found improved self-reported RTW rates [24]. Another Swedish study of a brief problem-solving intervention, which was offered to employees with stress-related symptoms through occupational health services and involved both employees and employers, found reduced registered sickness absence in the intervention group compared to control group [25]. Karlson et al. found that after a brief “convergence dialogue meeting” intervention, more individuals returned to part-time work during the study period compared to matched controls, however, there was no difference between the groups regarding full RTW after 1.5 years [26] nor at the 2.5 year follow-up [27]. In a similar Swedish study, a workplace dialogue reduced registered sickness absence in patients with exhaustion disorder [28]. However, there are also examples of recent clinical work-oriented interventions that failed to enhance RTW for patients with stress-related disorders [29, 30].

Thus, the current evidence suggests that the question of how to best help people who are on sick leave due to stress disorders to reintegrate into the workforce needs to be explored further. The present study aims to fill that knowledge gap. In recent years, rehabilitation coordinators (RCs) have been introduced in the Swedish health care system. Their role is to provide individual support to sick-listed patients and to coordinate with other public services and employers in the RTW process [31]. RCs work both in primary care and inpatient care, and since February 2020, all regions in Sweden are obliged to offer RC services to sick-listed patients that need it. The intervention evaluated in the present study was conducted during a period when rehabilitation coordination was just being established in primary care. It concerns an effect evaluation of the PRIMA intervention, a randomised controlled trial (RCT) designed to strengthen the cooperation between GPs and RCs and to involve employers in the rehabilitation process for patients with stress-related disorders [32].

## Aim

The aim of this study is to evaluate whether a systematic procedure involving collaboration between GPs, RCs and employers can reduce sick-leave days for patients with stress-related disorders during a 24-month follow-up period. An intervention group will be compared to treatment as usual (TAU).

## Methods

### Study design

The study took place in Region Västra Götaland, a county council of considerable size, encompassing nearly 20% of the Swedish population, with 200 public and private primary health care centres (PHCCs). The study was conducted at the Institute of Stress Medicine (ISM), Region Västra Götaland, and the Department of Work Science and Sociology, University of Gothenburg. PRIMA is part of the New Ways research programme, which is aimed at the identification and treatment of CMD and to provide support for persons with CMD so that they can continue to work, at the Section for Social Medicine and Public Health, Sahlgrenska Academy, University of Gothenburg. The study was registered on 16/01/2017 (ClinicalTrials.gov, NCT03022760).

### Recruitment, randomization and sample

The recruitment of PHCCs took place between January and October 2016. A total of 30 PHCCs were invited to participate from both public and private health care centres, and 22 accepted to participate (15 from public health care centres and seven from private health care centres). The centres were matched in pairs based on similarities in terms of size, ownership, socioeconomic conditions in the catchment area and the average proportion of enrolled patients that had a mental health diagnosis. The extent of RC resources was also taken into consideration. From each pair, one centre was randomly selected to the intervention group (*n* = 11) and the other to the control group (*n* = 11). Blinding was not possible in this study, as the allocation could not be concealed from either the case or control group.

The recruitment of patients took place at the 22 PHCCs between November 2016 and January 2018. The patients were invited to participate in the study at the intervention centres by their assigned RC. At the control centres, eligible patients were invited to participate by their GP.

Employed patients with a F43 diagnosis as the main diagnosis were eligible for participation, for summary of diagnostic criteria (see Table 1).

**Table 1:** Summary of F 43 diagnoses included in the present study

**F 43 Diagnoses included in the present study**	**Summary of diagnostic criteria**
F 43.0. Acute stress reaction	The main criteria for an acute stress reaction are the presence of intense fear, helplessness, or horror, and the development of symptoms that occur within one month of exposure to an extreme traumatic stressor. Symptoms may include persistent re-experiencing of the trauma, avoidance of reminders of the trauma, increased arousal, and negative alterations in cognitions and mood.
F 43.2. Adjustment disorder	The diagnostic criteria for adjustment disorder include the development of clinically significant emotional or behavioural symptoms in response to an identifiable stressor, within 3 months of the stressor's onset. Distress that is out of proportion with expected reactions to the stressor. The symptoms must cause clinically significant distress or impairment in social, occupational, or other important areas of functioning.
F 43.8.A Exhaustion Disorder	The diagnostic criteria for exhaustion disorder include persistent fatigue and exhaustion, accompanied by physical, cognitive and/or psychological symptoms, caused by stress exposure for at least 6 months. Additionally, the symptoms must be severe enough to cause clinically significant distress or impairment in social, occupational, or other important areas of functioning.
F 43.9. Reaction to severe stress	Reaction to severe stress diagnose is usually used for a short time of stress/crisis reaction, when the criteria for 43.0 Acute Stress Reaction, F43.2 Adjustment Disorder or F43.8A Exhaustion Syndrome are not met.

**Fig. 1:**
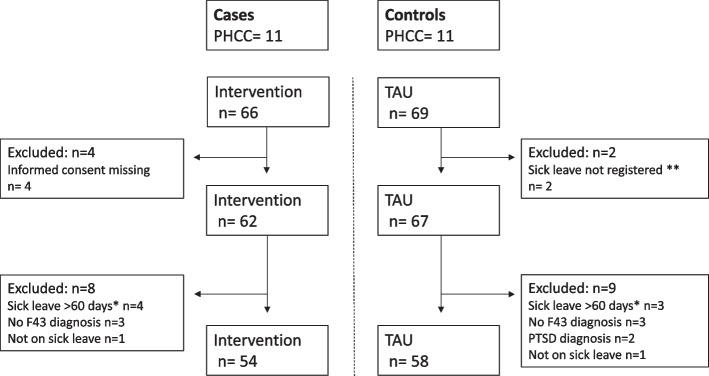
Flowchart of allocation of participant patients. PHCC = primary health care centres. TAU = treatment as usual. PTSD = post-traumatic stress syndrome. * Exclusion criteria: a former completed period of sick leave exceeding 60 days during the past three years. ** Sick leave was not registered in the sick leave insurance system

Patients with post-traumatic stress disorder (PTSD), patients who did not read or speak Swedish and patients who have had a sick-leave period of more than 60 consecutive days during the past three years were excluded. Patients seeking care for stress-related symptoms who met the inclusion criteria were included in the study after giving their informed consent. Treatment as usual (TAU), such as medical treatment or therapy, was offered to both cases and controls during the process. In total, 135 patients were recruited to the study, 66 to the intervention group and 69 to the control group. The allocation of participant patients is described in Fig. 1.

The final study sample included 112 participants, 54 participants in the intervention group and 58 participants in the control group (see Table 2). When included in the study, the participants were asked about their occupations, but there was a significant lack of responses. Among those who answered the question, there was a wide variety of occupations such as warehouse worker, seller or technician, with an overrepresentation of professions in education, healthcare, and social care.

**Table 2:** Study sample

	Intervention (*n*= 54)	TAU (*n*= 58)
**Gender**		
Female	46 (85%)	51 (88%)
Male	8 (15%)	7 (12%)
**Age**	42 (11.22 SD)	41 (11.88 SD)
**F43 diagnoses**		
F43.0 Acute stress reaction	4 (7%)	3 (5%)
F43.8 Other reactions of severe stress	3 (6%)	1 (2%)
F43.8A Exhaustion disorder	32 (59%)	27 (47%)
F43.9 Reaction to severe stress	15 (28%)	27 (46%)
**Sick leave days before inclusion**	39 (21.82 SD)	39 (28.33 SD)

*TAU* treatment as usual, *SD* standard deviation

### The intervention

The design of the intervention was based on the Person-Environment-Occupation model (PEO) that originates from occupational therapy, where occupational performance depends on the interaction between the person (P), the environment (E) and the occupation (O) [33]. The occupation relates to the work tasks the person is doing. The PEO-model suggests that interventions from the therapists should be directed to the individual, the work environment and the occupation (work tasks). By focusing on occupational performance and participation, therapists can facilitate a smoother transition back to the workplace and support the individual's well-being and successful reintegration into work-related activities.

**Fig. 2:**
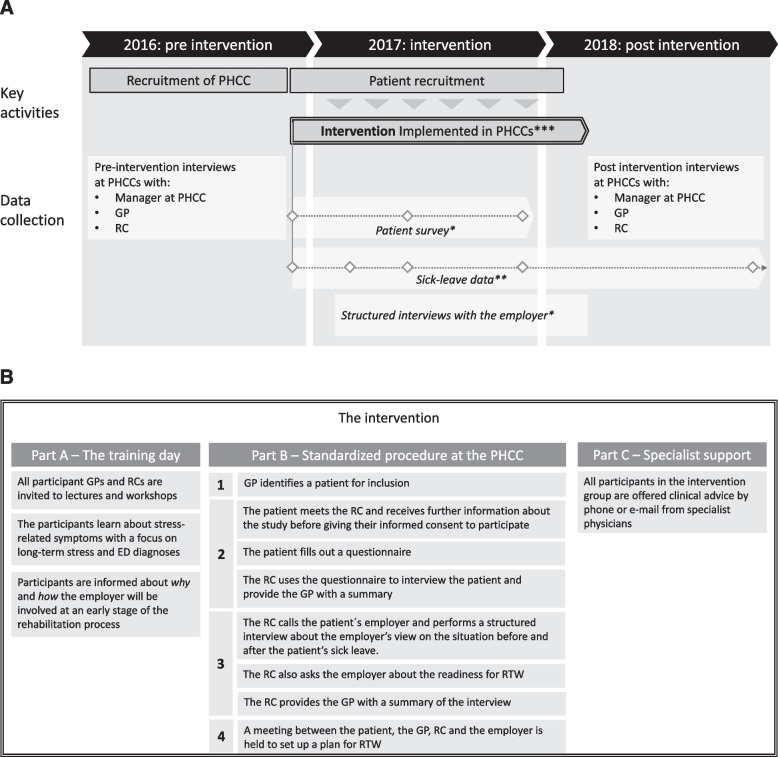
**a** Overview of the PRIMA project. GP = general practitioner, RC = rehabilitation coordinator, PHCC = primary health care centre. * intervention group only, **data used in the current study, *** see Fig. 2b. **b** Overview of the PRIMA intervention. GP = general practitioner, RC = rehabilitation coordinator, PHCC = primary health care centre, RTW = return to work

The intervention comprised a) a one-day training where all participant GPs and RCs were invited, b) a standardized procedure for GPs and RCs to follow after training and c) the opportunity for GPs to seek clinical advice from specialist physicians in the research group (see Fig. 2a and b).

The training day was intended to increase the knowledge of both GPs and RCs about stress-related disorders and RTW. The lectures and workshops were conducted by experienced physicians, a psychologist and an occupational therapist who were also researchers at the Institute of Stress Medicine (ISM) or at the University of Gothenburg. The participants learned more about stress-related symptoms with a focus on long-term stress and ED and the most common stressors associated with ED. Another topic covered was the working conditions, psychosocial work environment, and factors that facilitate or hinder return to work. The lectures also covered work ability, based on the PEO model, as well as how to evaluate a patient's work ability. Additionally, information about the social insurance system and regulations surrounding sick leave was provided. They were also informed why the employer should be involved at an early stage of the rehabilitation process and how the employer would be involved. A total of 76 GPs and 13 RCs underwent the one-day training; the number of participating GPs per centre varied from 2 to 13, however, all GPs working at the intervention PHCCs could contribute by referring patients for inclusion in the study. The standardised procedure (Part B in Fig. 2) contained four steps and was designed to involve the patient, the GP, the RC and the employer in the RTW process. Step one: when a GP identified a patient for inclusion, a meeting with the patient and the RC was arranged. Step two: the patient met the RC and received further information about the study before giving their informed consent to participate. The patient filled out a questionnaire containing questions on background characteristics, occupation, symptoms, work stressors and private life stressors, work ability, RTW self-efficacy, employer activities, RTW motivation and general health. The RC used the questionnaire to interview the patient and provided the GP with a summary. Step three: the RC called the patient´s employer and performed a structured interview about the employer’s view on the situation before and after the patient’s sick leave. The RC also asked the employer about the readiness for RTW, if the employer had any suggestions on what could be done at the workplace to enable RTW and if the employer took any steps to facilitate RTW. The structured interviews were inspired by a methodology developed at Umeå University [34] and the questions are available in Appendix 2. The GP was provided a summary of this interview. Step four: a meeting between the patient, the GP, RC and the employer was held to set up a plan for RTW. In the third part of the intervention, all participants in the intervention group were offered clinical advice by phone or e-mail from the specialist physicians at ISM during the intervention. The intervention has been described in detail in Bjork et al. [32].

### Intervention adherence

Among the two private and nine public intervention centres, the average proportion of enrolled patients that had a common mental health diagnosis in 2015 (the year the centres were recruited to the study) was 15% (range 13–18%). This was expected to provide a sound basis for patient recruitment. However, only five of the eleven intervention centres succeeded with protocol adherence. Together, RCs at these centres recruited 56 patients and successfully contacted 53 employers. In the six remaining centres, only 9 patients were recruited, and one employer contact was made. Post-intervention interviews with participant managers, RCs and GPs provide preliminary explanations for why centres failed or succeeded in their implementation. At the centres that succeeded, the RC had a clear role vis-à-vis the GPs, and routines for working with sickness certification and insurance medicine were well established before these centres engaged in the study. In other words, the compatibility between the intervention and pre-existing workflows was high. Second, centres that struggled with high workloads and staff turnover failed in their implementation. Here, both GPs and RCs were tasked with managing the daily operations, and their ‘readiness for change’ was low. However, these explanations should be explored further in future research.

### Outcome measures and statistical analyses

The primary outcome measures were the number of registered sick leave days for cases and controls at three, six, 12 and 24 months after inclusion. Data was retrieved from the Swedish Social Insurance Agency’s Micro Database for Analysing Social Insurance (MiDAS). In Sweden, sick leave benefits are granted for 25%, 50%, 75% or 100% of a working day, depending on how much the ability to work is reduced because of the diagnosis. Both gross days (number of sick days, regardless of extent of sick leave) and net days (number of sick days converted into whole sick days) were used as outcomes in this study.

The Mann–Whitney test was used to investigate the difference in gross and net days between the groups, with the level of significance set to *p* < 0.05. All analyses were conducted using the statistical package IBM SPSS Statistics 22.

## Results

Descriptive statistics for the intervention group and the control group (TAU) in gross and net days are presented in Table 3. The results show that the median number of registered gross sick leave days was lower for the control group at six, 12 and 24 months after inclusion, but the difference was not statistically significant. Gross days do not take the proportion of sick leave per day into account.

**Table 3:**
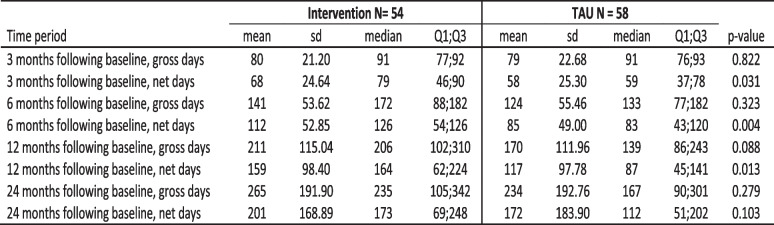
Comparisons of registered sick leave days between the intervention group and TAU

Mann–Whitney Test, sd = standard deviation, Q1;Q3 = first and third quartile

When looking at net days (i.e. the number of sick leave days converted into whole days), the advantage of the control group was further strengthened. At three months after inclusion, the control group had a median of 20 sick-leave days less than the intervention group (*p* = 0.031). At six months, this difference in median increased to a 43-day difference (*p* = 0.004). One year after inclusion, the difference in median between controls and cases was 77 net days (*p* = 0.013). However, two years after inclusion, no significant difference was seen between the groups.

## Discussion

The aim of the present study was to investigate whether the application of a standardised approach in a primary health care setting in patients with stress-related diagnoses could facilitate RTW. The findings point to the contrary: the PRIMA intervention appears to have protracted RTW times during the first year of follow-up. There was a significant difference in net sick leave days between cases and controls at three, six and 12 months after inclusion, where participants in the intervention group had more sick-leave days than those in the TAU group. At 24 months, the benefit of TAU for RTW could no longer be confirmed.

Previous attempts have highlighted the challenges of designing interventions with significant return to work (RTW) benefits. A recent review study focusing on RTW interventions for burned-out employees found inconclusive evidence regarding their effectiveness in facilitating RTW [35]. Only one study found a significant improvement in RTW (the intervention involved a convergence dialogue meeting between patients and supervisors to find effective solutions to facilitate RTW) [26, 27]. Two other studies involving contact with the employer (individual rehabilitation plan, gradual RTW and rehabilitation at the workplace) found no differences between the intervention and control groups [30, 36]. Finnes et al. [28] compared Acceptance and Commitment Therapy (ACT), a Workplace Dialogue Intervention (WDI), and a combination of ACT and WDI with TAU for patients with common mental disorders, including stress-related diagnoses. For the primary outcome, net sick leave days and work ability, none of the three treatment options performed better than TAU. Similar to our study, the WDI intervention increased sick leave days. Holmgren et al. [37] also found no evidence that an intervention based on the early identification of work-related stress in non-sick-listed employed persons who seek primary health care due to psychical and mental health symptom, paired with a training session to increase GP awareness, could reduce sick leave days compared to TAU. Several explanations for these results are discussed in these papers, some of which are also relevant to this study. In the Swedish national decisions support for insurance medicine, the recommendations for sick leave for ED diagnosis in Sweden is six to 12 months of sick leave or sometimes longer for severe cases. Furthermore, the sick leave regulations for all diagnoses in Sweden suggest RTW within one year [38]. This recommendation likely had an effect on the results of the present study as well: patients are encouraged or sometimes forced to full RTW within one year, even if they have not fully recovered. However, if more structured plans for RTW are made with important stakeholders, the Swedish Social Insurance Agency may be more willing to accept sick leave periods that extend beyond one year. This could have enabled longer sick leave periods in the intervention group.

Another possible explanation for the results could be, as mentioned in Holmgren et al. [37], that a short training session is not sufficient to produce desired behavioural changes among participants. Bakker et al. [39] also found limited effects on GP adherence to new routines after a single training day focusing on interventions for stress-related patients. In the current case, however, we would instead suggest that the training had an important impact. The main focus of the training day was to increase participant knowledge of stress-related diagnoses and the association between work and stress. The part of the training day that was directed primarily at the GPs focused on long-term stress and ED. Information was provided to inform GPs that for some patients in this group, a longer sick leave period is sometimes necessary. A possible explanation for why the patients in the control group had less sick leave days than patients in the intervention group could thus be that the 76 GPs who underwent training and then went back to their PHCCs to recruit patients to the study were more prone to accept a longer rehabilitation process and longer periods of sick leave.

The second component of the intervention was the standardised process, where contact with the employer was established and a plan for RTW was elaborated between the patient, the employer, the RC and the GP. It is plausible that this component also prolonged, rather than reduced the time to RTW. The employer´s readiness for RTW may not be in line with the patient´s readiness, and this could potentially prolong the process. Seing et al. [40] found that the employer had a considerable role in the RTW process and often decided whether or not the employee could RTW.

Since we only use sick leave days as an outcome, we have no insight into the patients’ wellbeing after the intervention. It is possible that even though the intervention failed to reduce time to RTW, the elaborated plan for RTW, which was agreed upon with the employer, provided a safe environment that helped reduce symptoms in the intervention group. Glise et al. found [13] that 31% of patients with ED were still clinically exhausted after seven years, although almost 90% were not on sick leave. These results indicate that RTW does occur even when patients have persistent symptoms. To enable this, it is likely that adjustments need to be made during the RTW process, including both individual coping strategies and adjustments at work. A recent study found that approximately two thirds of the participants with ED made some kind of change at work due to their disease after seven years, such as change of workplace, work tasks or reduced working hours [41]. It can also be important to consider how patients with ED function at work when they RTW.

Van Hess et al. [42] provided insight into different aspects relevant to patients with CMD that promote work performance and the ability to persevere in the workplace, such as organisational climate, social support and coping strategies that enable employees with CMD to participate at work. Patients with CMD may perceive that their workflow is affected and feel disconnected from work [43]. Danielsson et al. found that patients with CMD use different cognitive, behavioural and social strategies to enable work performance [44].

The intervention in this study was based on the PEO model, which emphasizes the importance of considering not only individual factors that affect RTW, but also the work environment and occupation. Previous research has shown that work-related stress is associated to the psychosocial work environment, such as low co-worker support, low supervisor support, low procedural justice, low relational justice and a high effort–reward imbalance [45, 46]. The psychosocial work environment is also of importance in the RTW process [47]. Additionally, it may not be possible to implement certain adjustments that could facilitate RTW, such as flexible working hours, working from home, or a quieter workspace, depending on the nature of the occupation.

An interesting finding was that the difference between the groups was no longer significant after 24 months. This raises the question whether the results would be different if there was a longer follow-up period, i.e., if the intervention would have promoted more sustainable RTW. A follow-up period of two years can be considered to be quite extensive in comparison to similar studies but still too short to detect differences in relapse into sickness absence between cases and controls. A strength of the present study is that the data on sick leave was retrieved from official registers and not self-reported, reducing the risks for recall bias and drop-out.

### Limitations

We would like to explore the possibility that TAU is adequate on its own. This has been confirmed in other studies on CMD in primary health care. Kivi et al. [48] found no support for the introduction of Internet-based Cognitive Behaviour Therapy (ICBT) beyond TAU as a treatment option in primary health care for patients with mild to moderate depression. The PRIMA intervention was conducted when the function of RCs had recently been introduced. At the time, there was a great deal of discussion within the field of rehabilitation about the importance of employer involvement [49, 50]. There were also active discussions about the need for the Swedish Social Insurance Agency to take responsibility by convening meetings between sick-listed patients, employers and GPs. Hence, it is possible that the PHCCs in the control group were elaborating their methods for employer involvement during this time, so that the difference between the intervention’s components and TAU were not as pronounced.

In addition to the problems that have already been discussed – i.e. issues with the intervention content, lack of data on symptom development and limited knowledge about what happened in the control group – there is one more important limitation to be raised. It would have been desirable to make sub-group comparisons within the intervention group in order to deepen our knowledge about the mechanisms involved [51], however, a limited sample size (54 participants) did not permit such analyses. It is plausible that the use of fewer inclusion criteria and a less standardised work procedure would have made it easier for the participant GPs and RCs to include more patients and follow through with the protocol. Balancing scientific rigour (e.g. a well-defined population and protocol adherence) with the participants’ wish for simplicity and adaptation to local needs is a challenging task, which is often raised in the implementation of research in care settings [18]. As indicated by the post-intervention interviews, the involvement of workplaces that are ready for change in terms of organisational prerequisites (e.g. solid structures and routines, staff continuity and a good psychosocial work environment) is also paramount. A good fit between the intervention’s components and the setting where these are to be implemented has been highlighted in both general [52] and primary-care specific [53] implementation frameworks, and the experiences from the current study support this. Although substantial efforts were made to select suitable health care centres for the study, even higher thresholds for inclusion would have been desirable. Regardless of this, the sample size was large enough to explore and detect differences between cases and controls in RTW, which was the main purpose of the current study and of the intervention.

## Conclusion

The aim of this randomised controlled trial was to evaluate whether a systematic procedure of collaboration between general practitioners and rehabilitation coordinators, which involved the employer in the rehabilitation of sick-listed patients with stress-related disorders, could reduce the number of sick-leave days during a 24-month follow-up period. Contrary to what was expected, the intervention prolonged the RTW time, measured as net sick leave days at three, six, and 12 months after inclusion, as compared to treatment as usual. At 24 months, no significant difference between cases and controls was found.

### Supplementary Information

**Additional file 1. **A summary of F 43 diagnoses included in the present study.

**Additional file 2. **Questions for employer interviews.

## Data Availability

The datasets generated and analysed during the current study is available from the corresponding author on reasonable request.

## Abbreviations

RTW: Return To Work
GPs: General Practitioners
RCs: Rehabilitation Coordinators
PHCCS: Primary Health Care Centres
TAU: Treatment As Usual
ED: Exhaustion Disorder
CMD: Common Mental Disorders
PEO: Person-Environment-Occupation model
ISM: Institute of Stress Medicine
ACT: Acceptance and Commitment Therapy
WDI: Workplace Dialogue Intervention
ICBT: Internet-Based Cognitive Behaviour Therapy
