# Supplementary material for: Does employer involvement in primary health care enhance return to work for patients with stress-related mental disorders? a cluster randomized controlled trial
Source: BMC Prim Care. 2023 Sep 20;24:195. doi: 10.1186/s12875-023-02151-0 (PMC10512560; doi:10.1186/s12875-023-02151-0)
Supplement: Supplementary file 1 — Additional file 1. A summary of F 43 diagnoses included in the present study. [file 12875_2023_2151_MOESM1_ESM.docx]

**A summary of F 43 diagnoses included in the present study**

| **F 43 Diagnoses included in the present study** | **Summary of diagnostic criteria** |
| --- | --- |
| F 43.0. Acute stress reaction | The main criteria for an acute stress reaction are the presence of intense fear, helplessness, or horror, and the development of symptoms that occur within one month of exposure to an extreme traumatic stressor. Symptoms may include persistent re-experiencing of the trauma, avoidance of reminders of the trauma, increased arousal, and negative alterations in cognitions and mood. |
| F 43.2. Adjustment disorder | The diagnostic criteria for adjustment disorder include the development of clinically significant emotional or behavioural symptoms in response to an identifiable stressor, within 3 months of the stressor's onset. Distress that is out of proportion with expected reactions to the stressor. The symptoms must cause clinically significant distress or impairment in social, occupational, or other important areas of functioning. |
| F 43.8. A Exhaustion Disorder | The diagnostic criteria for exhaustion disorder include persistent fatigue and exhaustion, accompanied by physical, cognitive and/or psychological symptoms, caused by stress exposure for at least 6 months. Additionally, the symptoms must be severe enough to cause clinically significant distress or impairment in social, occupational, or other important areas of functioning |
| F 43.9. Reaction to severe stress | Reaction to severe stress diagnose is usually used for a short time of stress/crisis reaction, when the criteria for 43.0 Acute Stress Reaction, F43.2 Adjustment Disorder or F43.8A Exhaustion Syndrome are not met. |
