# Supplementary material for: Does employer involvement in primary health care enhance return to work for patients with stress-related mental disorders? a cluster randomized controlled trial
Source: BMC Prim Care. 2023 Sep 20;24:195. doi: 10.1186/s12875-023-02151-0 (PMC10512560; doi:10.1186/s12875-023-02151-0)
Supplement: Supplementary file 2 — Additional file 2. Questions for employer interviews. [file 12875_2023_2151_MOESM2_ESM.docx]

**Questions for employer interviews**

1. To what extent do you think that the employee's sick leave is caused by conditions at work?
2. To what extent do you think that the employee's sick leave is caused by circumstances outside of work?
3. As an employer, did you do something to facilitate the work situation before the actual sick leave?
4. Did the employee try to bring about any changes before the actual sick leave?
5. Do you have any suggestions on what could be done in order for the employee to return to work?
6. Have you as an employer done something to facilitate return to work?
7. Is there any possibility for vocational rehabilitation at the workplace?
8. Has the employer procured occupational health services that provide services for rehabilitation?
9. Are you aware of whether the employee has used occupational health services during the current sick leave?
